# Valorization of *Chlorella thermophila* biomass cultivated in dairy wastewater for biopesticide production against bacterial rice blight: a circular biorefinery approach

**DOI:** 10.1186/s12870-023-04579-z

**Published:** 2023-12-15

**Authors:** Satya Sundar Mohanty, Kaustubha Mohanty

**Affiliations:** 1 School of Energy Sciences and Engineering, Indian Institute of Technology Guwahati, Assam, India; 2 Department of Biotechnology, Karunya Institute of Technology and Sciences, Coimbatore, Tamil Nadu India; 3 Department of Chemical Engineering, Indian Institute of Technology Guwahati, Assam, India

**Keywords:** Biocrude, Biopesticide, Chlorella, Dairy wastewater, Remediation

## Abstract

Biopesticides offer a sustainable and efficient alternative to synthetic pesticides, providing a safer and more eco-friendly solution to pest management. The present work proposes an innovative approach that integrates crop protection and wastewater treatment using thermophilic microalgal strain *Chlorella thermophila (CT)* cultivated in nutrient-rich dairy wastewater as a growth medium. The microalgae was cultivated mixotrophically and was able to reduce both organic carbon as well as nutrient load of the dairy wastewater efficiently. The integrated circular biorefinery approach combines biomass cultivation, extraction of biopesticide compounds, and conversion to biocrude. The antimicrobial activity of the biopesticidal extracts against *Xanthomonas oryzae* and *Pantoea agglomerans*, the causative agent of bacterial rice blight, is assessed through in vitro studies. The biomass extract obtained is able to inhibit the growth of both the above-mentioned plant pathogens successfully. Mass spectroscopy analysis indicates the presence of Neophytadiene that has previously been reported for the inhibition of several pathogenic bacteria and fungi. Several other value-added products such as linoleic acid and nervonic acids were also been detected in the microalgal biomass which have extremely high nutraceutical and medicinal values. Furthermore, the study investigates the potential for co-production of biocrude from the biorefinery process via hydrothermal liquefaction. Overall, the findings of this present work represent an innovative and sustainable approach that combines wastewater treatment and crop protection using microalgal biomass.

**Supplementary Information:**

The online version contains supplementary material available at 10.1186/s12870-023-04579-z.

## Introduction

Rice is a crucial food crop for more than 65% of the world's population, especially in many Asian countries where it is an integral part of the culture and economy. Access to rice has significant impacts on food and livelihood security, as well as political stability in rice-dependent developing economies [1]. India and China are the largest producers of rice, contributing over half of the global production of 500 million tonnes annually on approximately 160 million hectares of land [2]. However, to meet the demands of the increasing global population, rice production needs to increase by nearly 42%, which is a challenging task due to various biotic and abiotic factors, including plant diseases which pose one of the biggest threats [3].

Bacterial leaf blights (BLB) have become a major concern for farmers and agricultural industries worldwide due to the enormous economic losses caused to both agricultural and horticultural crops. This devastating foliar disease is prevalent in various regions including Asia, Africa, Australia, western coast of Latin America, and Caribbean countries, and is caused by bacterial pathogens such as *Pantoea agglomerans* and *Xanthomonas oryzae pv. Oryzae* [4–6]. BLB not only reduces grain yield but also severely affects the quality of the straw, which is used as fodder [7]. Yield losses due to BLB can be as high as 80%, as reported in several studies [3, 8]. Outbreaks of BLB are periodically observed in various regions of Asia, particularly South Asia, and have been reported in studies from different countries, including but not limited to, Korea, India, and Malaysia [9].

The threat to global food security posed by BLB highlights the need for effective control measures. While chemical control using antibiotics or pesticides has been used for many years, their indiscriminate use has raised concerns about safety, practicality, antibiotic resistance, high cost, and adverse effects on the environment and soil fertility [10]. Therefore, a more sustainable and cost-effective approach is required. Biological control, in which living organisms are used to suppress the density and activity of plant pathogens, offers a promising alternative or supplement to agrochemicals for disease management [11]. These organisms, also known as biological control agents or antagonists, can effectively suppress plant pathogens, making biological control a valuable strategy for sustainable agriculture [12].

In recent times, microalgae and cyanobacteria have gained attention due to their adaptability and diversity. These organisms are not only a potential source of valuable bioactive compounds such as biofuels and food supplements but also have the ability to positively influence plant productivity. Apart from enhancing soil fertility, seed germination, plant growth, nutritional value, and crop yield, these microorganisms do not compete with other microorganisms present in the plant-soil system but rather support beneficial activities attributed to rhizo-microbiota [12]. The bioactive compounds produced by microalgae and cyanobacteria include alkaloids, indoles, fatty acids, siderophores, amides, and antimicrobial substances such as lipopeptides and polyketides [13, 14]. In addition to these properties, microalgae also contain a variety of secondary metabolites that can act as biopesticides, providing protection to the plant against diseases caused by pathogens. Several studies have reported the isolation of antimicrobial compounds from microalgae, including Scenedesmus, a green microalga found in freshwater environments. Mendiola et al. (2007) reported the isolation of a polysaccharide with antimicrobial activity from *Scenedesmus obliquus*, while Lee et al. (2020) identified a novel cyclic peptide with antibacterial activity from *Scenedesmus sp*. [15, 16]. More recently, a study conducted by Alsenani et al. (2020) demonstrated the antifungal activity of extracts from the microalga *Chlorella vulgaris* against the fungal pathogen *Fusarium oxysporum* [17]. The authors found that the extract inhibited the growth of the fungus and reduced its ability to cause disease in tomato plants. The authors found that the extract was effective in reducing disease severity and stimulating plant growth, suggesting that microalgae-based products may have dual benefits for plant health. These findings suggest that microalgae may offer a promising source of biocontrol agents for the management of plant diseases.

However, the use of microalgae-based products for plant pathogen control is still in its early stages, and further research is needed to determine the potential of these agents for agricultural applications. Singh et al. (2016) noted that the use of microalgae as biocontrol agents presents several challenges, including the identification of the most effective strains, optimization of growth conditions, and development of appropriate extraction and formulation methods [18]. Moreover, the mode of action of microalgae-based biocontrol agents remains largely unknown, and additional studies are needed to elucidate the mechanisms by which these agents provide protection to the plant.

In the present study we focused on the mixotrophic cultivation of the microalgae *Chlorella thermophila (CT)* in synthetic dairy wastewater and evaluate the bioactive potential of the cell extracts against the plant pathogens *Xanthomonas oryzae* pv. *Oryzae* and *Pantoea agglomerans.* For the present study, the strain was selected due to its efficient reduction in Chemical Oxygen Demand (COD) and nutrient from the wastewater and potential biopesticide activity. This will establish the groundwork for future research to develop an eco-friendly and sustainable strategy for coupling industrial wastewater treatment along with management of harmful plant pathogens.

## Experimental methodology

### Microorganisms and culture conditions

In the present study, the microalgae strain *Chlorella thermophila* (MF179624) has been investigated for its ability to remediate the dairy wastewater and production of biopesticide. To culture the microalgal strain, the inoculum was grown in the inorganic BG-11 medium with continuous aeration and a pH of 7.2 ± 0.2. The light intensity and temperature were set at 100 μmol/m2s and 28 ± 2 °C, respectively, with a photoactivity ratio of 16:8 h of light and dark period. The bacterial strains *Pantoea agglomerans* (MTCC 6720) and *Xanthomonas oryzae pv. oryzae* (MTCC 11105) were obtained from the CSIR – Institute Of Microbial Technology (IMTECH), Chandigarh, India, and were maintained on nutrient medium.

### Batch studies

In this study, the microalgal strain was grown using synthetic dairy wastewater (SDWW) as the culture medium. The composition of the SDWW was obtained from the literature [19]. The initial physico-chemical characteristics of the synthetic dairy wastewater was COD: 3600 mg/L; Nitrate: 160 ± 3 mg/L; Phosphate: 180 ± 2.1 mg/L. The experiments were carried out in 2000 mL wide mouth screw cap bottles containing 1500 mL of synthetic dairy wastewater (SDWW) along with 10% inoculum. The temperature was maintained at 28 ± 2 °C with the light intensity of 7400 lx for a photoperiod of 16:8 h (light: dark). The culture medium was sparged continuously using an aquarium aerator for the homogenous mixing of the components. It was evaluated on a regular interval to determine the biomass growth, and residual nutrient and COD concentration of the medium. All the experiments were performed in triplicates and the mean value of all were presented in the graphs.

### Growth studies and biochemical characterization

The biomass growth quantification involved harvesting 1 mL of the culture at 5000 RPM for 10 min, followed by rinsing with distilled water and resuspending it in phosphate buffer saline. The biomass concentration was determined using spectrophotometer at 680 nm (Thermo Fisher Scientific, USA). The biomass productivity (mg/l/d) was calculated using the following formula [20]:

1 $$Biomass\, Productivity = \left\{{}^{\left({C}_{t} - {C}_{0}\right)}\!\left/ \!{}_{\left(d - {d}_{0}\right)}\right.\right\}$$

Where C_0_ and C_t_ represent the dry cell weight (DCW) of the initial (d_0_) and final (d) day of the experiment, respectively.

The elemental composition of the cells was analyzed by an elemental analyzer (Perkin-Elmer Thermo Scientific Flash 2000). Furthermore, the biomass was assessed for its protein, carbohydrate, and lipid content using the Lowry method, modified phenol–sulfuric acid method, and modified Bligh and Dyer extraction method, respectively, as described in the literature [21].

### COD Reduction and nutrient consumption analysis

The reduction in the Chemical Oxygen Demand (COD) of the synthetic dairy wastewater by the microalgal strain was assessed as per the available protocol [22]. The consumption of nutrients was estimated by analyzing the residual nitrate and phosphate content of the culture medium, following the recommended protocols by APHA [23].

### Antagonism bioassays against phytopathogens

Antagonism bioassays against phytopathogens were conducted using the microalgal extract following protocols established in the previous studies [24, 25]. In brief, the microalgal biomass was harvested and homogenized with glass beads under controlled agitation. The resulting supernatant was separated, mixed with different solvents such as n-Hexane, chloroform, and methanol. The mixture was further concentrated using a rotavapor [26]. To assess the bactericidal activity of the microalgal extract against phytopathogens, the well diffusion experiment was employed [25, 27]. Autoclaved distilled water containing the organic solvents served as the negative control. The inhibition index (I%) was calculated using the following formula [25]:

2 $$I \left(\%\right) =100 - \left\{M - \frac{N -8}{90}\right\} \times 100$$

Where M represents bacterial growth diameter in the absence of any antibacterial compound (which, in the present study, corresponds to 90 mm petri plates) and N represents the zone of inhibition in millimeters.

For further analysis, the microalgae biomass extracts underwent GC–MS analysis using a Perkin Elmer instrument equipped with a capillary column (ID: 0.25 mm, length: 60 m, film thickness: 0.25 µm). The capillary column used was 'Elite-5MS'. The identification of individual mass spectra from the unknown samples was accomplished by comparing them to the mass spectra available in the NIST Library.

### Production of biocrude oil from post-extraction microalgal biomass

The hydrothermal liquefaction (HTL) process was employed to convert the *Chlorella thermophila* biomass obtained after extraction of biopesticide into biocrude oil following a standard protocol [28]. Initially, a 10% (w/v) aqueous slurry was prepared using the microalgae biomass. The slurry was then introduced into a 100 mL autoclave reactor (Parr, USA). To maintain an inert working atmosphere within the reactor, it was purged with high-quality nitrogen gas for a duration of 2 min. Subsequently, the reactor was pressurized to 2 MPa to check for any leaks and establish the initial pressure. The reaction conditions included a temperature range of 300–350 °C with a heating rate of 5 °C per minute, a pressure range of 8.5–12 MPa (autogenous pressure), a residence duration of 30 min (excluding the preheating period), and continuous agitation at 400 rpm. These parameters were carefully controlled to ensure the efficient progress of the hydrothermal liquefaction process. After each HTL experiment, the product was separated by quenching the reactor in a room-temperature water bath and then depressurizing it. The organic mixture was then diluted and extracted using 10 mL of dichloromethane (DCM) in the reactor. To separate each phase, the product mix was transferred to centrifuge tubes and spun at 4500 rpm for 15 min. Each phase was quantified by evaporating the DCM with a rotary evaporator (Rotavapor® R-210, Buchi, Switzerland). Previously, a detailed experimental procedure was provided [29]. The moisture-free biocrude was gravimetrically measured. The biocrude samples were kept at 20 °C for future examination.

### Estimation of cellular bioenergy and HHV value

The biomass energy or bioenergy (kJ) of microalgae can be calculated using the following equation:

3 $$\mathrm{Bionergy}\left(\mathrm{kJ}\right)=\left(\mathrm{Weight}\;\mathrm{of}\;\mathrm{Carbohydrate}\;\left(\mathrm g\right)\times15.7\mathrm{kJ}/\mathrm g\right)+\left(\mathrm{Weight}\;\mathrm{of}\;\mathrm{Lipid}\;\left(\mathrm g\right)\times37.6\mathrm{kJ}/\mathrm g\right)+\left(\mathrm{Weight}\;\mathrm{of}\;\mathrm{Protein}\;\left(\mathrm g\right)\times16.7\mathrm{kJ}/\mathrm g\right)$$

In this equation, the weight of carbohydrate, lipid, and protein are the amounts of these primary metabolites present in the microalgae biomass. The energy released during the full oxidation of each macromolecule (15.7 kJ/g for carbohydrate, 37.6 kJ/g for lipid, and 16.7 kJ/g for protein) is then multiplied by their respective weights, and the sum of these products gives the total biomass energy or bioenergy in kilojoules (kJ). This value represents the amount of energy that can potentially be obtained from the complete combustion of the macromolecules present in the microalgae biomass.

The higher heating value (HHV) of biomass can be calculated using the method developed by Channiwala and Parikh, which takes into account the elemental composition and ash content of the biomass [30]. The HHV is a measure of the energy content of the biomass when it is completely burned. The formula to calculate the higher heating value (HHV) is as follows:

4 $$\mathrm{HIV }=0.349\mathrm{C }+1.178\mathrm{H }-0.1034\mathrm{O }+0.151\mathrm{N }+0.1005\mathrm{S }-0.0211\mathrm{A}$$

Where: C = Carbon content; H = Hydrogen content; O = Oxygen content; N = Nitrogen content; S = Sulfur content; A = Ash content. Each element's content is expressed as a percentage of the total weight of the biomass. This method provides an estimate of the energy value of the biomass based on its elemental composition and ash content and is commonly used in bioenergy and biomass-related research.

## Results and discussion

### Growth study of *Chlorella thermophila* (MF179624)

The microalgal strain *Chlorella thermophila* (MF179624) was cultivated mixotrophically in simulated dairy wastewater (SDWW). The microalgae biomass increased from 0.25 g/L (initially) to 2.1 g/L at the time of harvesting which is almost 25% more than that obtained using the inorganic BG-11 medium. however, it was observed that during the initial two days of the experiment, the microalgal growth experienced a lag phase where the DCW of the microalgal biomass increased to only 0.34 g/L from 0.25 g/L. However, afterward vigorous growth with progressive settling was observed indicating the adaptation of the microalgal species to the new environment [31]. Regular monitoring of growth characteristics revealed that the green hue of the microalgal culture intensified over time and this is well correlated with the treatment of the dairy wastewater in terms of nutrient and organic carbon removal. The biomass productivity was significantly higher in SDWW, with 175 mg/L/d as compared to 141 mg/L/d in BG-11. The findings suggest that SDWW is a more effective nutritional source for the microalgal strain, which may account for the greater biomass productivity and potentially lead to improved remediation efficiency and carbon biofixation. A previous study on *Monoraphidium sps*. grown in dairy wastewater also reported similar results, with a biomass yield of 1.47 g/L [28].

**Fig. 1:**
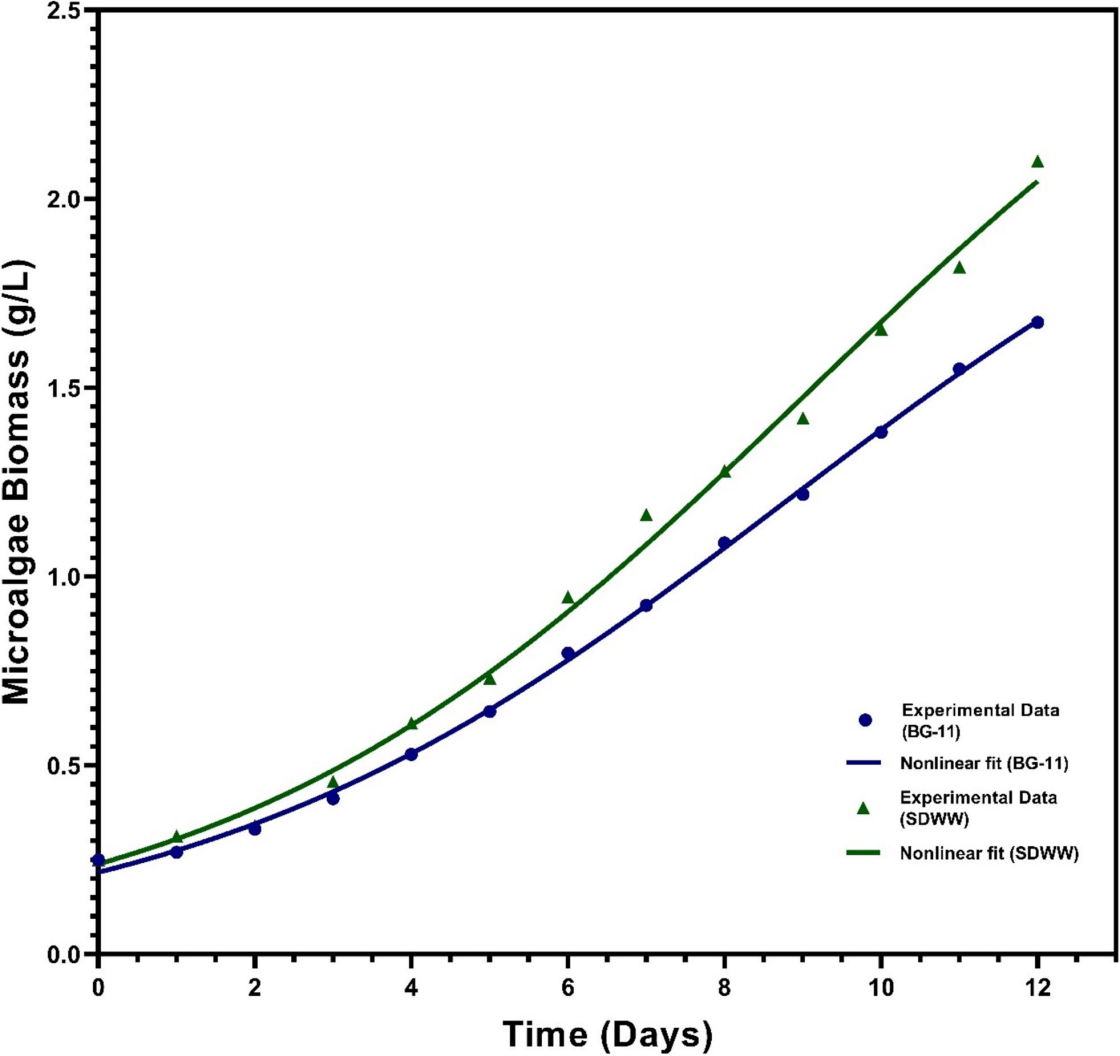
Growth Curve of *Chlorella thermophila* in SDWW

The growth period, characterized by the peak growth rates, was assessed and validated using two non-linear mathematical models: the Logistic and Gompertz models. These models are commonly utilized to depict rapid population expansion in organisms. The fitting of the model and the comparative study was carried out using the GraphPad Prism software 8.0.1. Eventhough both the models seem to fit the experimental data decently, based on the Akaike information criterion (AIC) value and the Anderson–Darling test, the Logistic model demonstrated superior fitting of the microalgae growth curves compared to the Gompertz model (Fig. 1). The AICc value of Gompertz Model fit is -61.32 as compared to -65.20 for the Logistic model fit. While the true relationship is impossible to know, the model with the lowest AIC score is considered to be the model that best represents the true relationship with the given data [32]. This is corroborated by the Anderson–Darling Normality Test of both the model fits. This goodness-of-fit test allows to control the hypothesis that the distribution of the observed variable in a sample follows a certain theoretical distribution. In particular, it allows us to test whether the empirical distribution obtained corresponds to a normal distribution [33]. Lower the AD value, better is the model fit. The AD value for the Logistic model is 0.1677 as compared to the Gompertz model with the AD value of 0.2277 thus reiterating the above-mentioned fact that eventhough both the model fits well with the experimental data, the Logistic model has a superior fitting with the available set of experimental data. The R^2^ values, surpassing 0.99, indicated that the model effectively captured over 99% of the variability in microalgae biomass concentration. The logistic growth model is a widely used mathematical framework to describe the population growth of various organisms. It accounts for the fact that as a population grows, it encounters limitations such as resource availability and space, causing its growth rate to slow down and eventually stabilize. The logistic growth model can be represented by the following equation:

5 $$\mathrm{Y }= {}^{{\mathrm{Y}}_{\mathrm{M}} \times {\mathrm{Y}}_{\mathrm{O}}}\!\left/ \!{}_{\left(\left({\mathrm{Y}}_{\mathrm{M}} - {\mathrm{Y}}_{\mathrm{O}}\right) *\mathrm{exp }\left(-\mathrm{ k }*\mathrm{x}\right) + {\mathrm{Y}}_{\mathrm{O}}\right)}\right.$$

Where: Y_0_ is the starting population (same units as Y), Y_M_ is the maximum population (same units as Y), k is the rate constant (inverse units of X), 1/k is the X coordinate of the first inflection point. The model fit infers that the maximum carrying population for the growth of *Chlorella thermophila* using only dairy wastewater as the cultivation medium could be as high as 3.018 g/L with an initial inoculum size of just 0.2365 g/L and intrinsic growth rate of 0.2688 day^−1^ with an initial lag phase of 3.720 days.

In this context of microalgae growth, the logistic growth model can be used to describe how the population of microalgae changes over time as they grow in a nutrient rich dairy wastewater medium. Initially, when the population size is small compared to the carrying capacity, the population grows exponentially, similar to the unrestricted growth phase. As the population approaches the carrying capacity, the growth rate slows down due to resource limitations, leading to a more gradual increase in population size. In the early stages of cultivation, when resources are abundant, microalgae population increases rapidly. However, as the culture becomes denser and resources become limited, the growth rate starts to decrease until it levels off when the carrying capacity is reached. By fitting experimental data to the logistic growth equation, researchers can estimate parameters like the intrinsic growth rate and carrying capacity, which are essential for optimizing microalgae cultivation strategies.

Microalgae cultivation is significantly influenced by pH, as it plays a crucial role in regulating various aspects of microalgal cell metabolism [34]. It also affects the redox behaviour, availability and solubility of carbon dioxide and nutrients for the microalgal growth. Therefore, maintaining an optimal pH range is crucial for microalgal growth and productivity. In the present study, the initial pH of the experimental setup was maintained at 7.2 which increases to 10.5 ± 0.2 during the treatment. The shift in pH towards basicity is often observed in self-buffering systems of microalgae cultivation, which can be attributed to the experimental conditions and environmental factors. This self-buffering mechanism helps to stabilize the pH and maintain a suitable growth environment for microalgae [19]. This pattern is anticipated, as the pH in photosynthetic systems is known to rise due to the generation of hydroxide ions resulting from the release of oxygen molecules, which is a byproduct of the photosynthesis process. The alkaline pH level facilitates the utilization of carbon for the synthesis of metabolites, which is directly correlated to the biomass concentration and the degradation of organic substrates [35]. This highlights the importance of pH control in optimizing the yield of desired metabolites and biomass in microalgae cultivation.

### Nutrient removal and dairy wastewater treatment using *Chlorella thermophila*

**Fig. 2:**
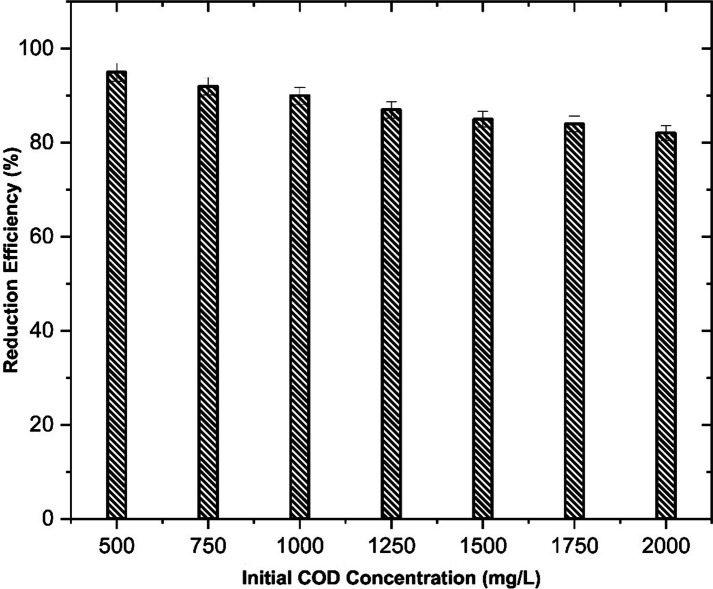
COD Reduction efficiency of *Chlorella thermophila* in SDWW

Dairy industry wastewater is characterized by high organic load, nutrients, and suspended solids, making its use as a cultivation medium for microalgae is a sustainable and low-cost alternative for biomass production. In the present study, we investigated the ability of the microalgal strain *Chlorella thermophila* (MF179624) for its ability to assimilate the organic carbon and the nutrient available for the treatment of the dairy wastewater and biomass yield. The strain was subjected to varying concentration of the dairy wastewater to determine the effect of initial COD concentration on the reduction efficiency as illustrated in Fig. 2. The results obtained indicate a negative correlation between the COD concentration and the reduction efficiency, with a decrease in efficiency as the COD concentration increases. This may be due to the fact that the microalgal strain is unable to withstand such high COD concentration and it confers an adverse impact on the biomass productivity. However, the microalgal strain is not only able to tolerate an initial COD concentration of as high as 2000 mg/L but is also able to reduce up to 82% of the initial COD concentration within a span of 12 days. The results obtained in the present study makes the strain *Chlorella thermophila* a promising candidate for the treatment of wastewater from dairy industry which usually contains such high organic carbon load.

**Fig. 3:**
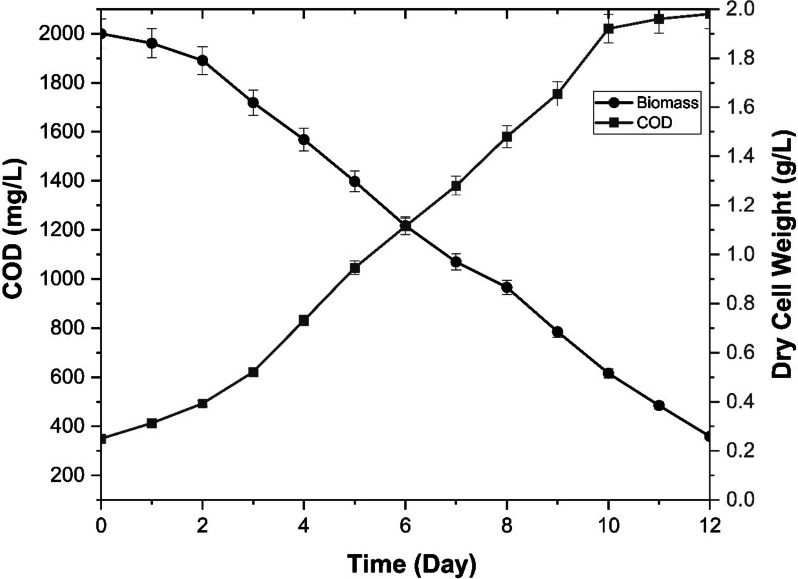
Biomass yield and COD Reduction Profile of *Chlorella thermophila* in SDWW

Figure 3 illustrates the correlation between COD reduction effectiveness and the cell biomass yield of the microalgal species. The figure infers that the microalgal strain is not only able to reduce the organic carbon load of the wastewater but also is able to utilize it to acquire higher biomass yield. While the biomass yield incase of the inorganic BG-11 medium is 1.5 g/L, the biomass yield for the dairy wastewater was found to be almost 40% more than it for the same amount of cultivation time. Similar trends have been observed in a previous study where the microalgal strain Monoraphidium sp. KMC4 exhibited a gradual decrease in COD reduction efficiency with increasing initial COD concentrations (from 480 mg/L to 3840 mg/L) [28]. Comparable results have been reported for other microalgal species such as *Coelastrella sp.*, which demonstrated a COD reduction efficiency of up to 69% in dairy wastewater [36]. However, Divya Kuravi and Venkata Mohan, (2022) reported higher reduction up to 75% of the initial COD concentration in dairy wastewater [19]. Hadiyanto et al.,(2019) reported the application of Spirulina platensis for the treatment of nutrient rich cassava processing wastewater where the microalgal strain is able to reduce upto 67% of the initial COD concentration and simultaneously generate bioelectricity of about 14.47 ± 0.7 mW·m^−2^ [37].

**Fig. 4:**
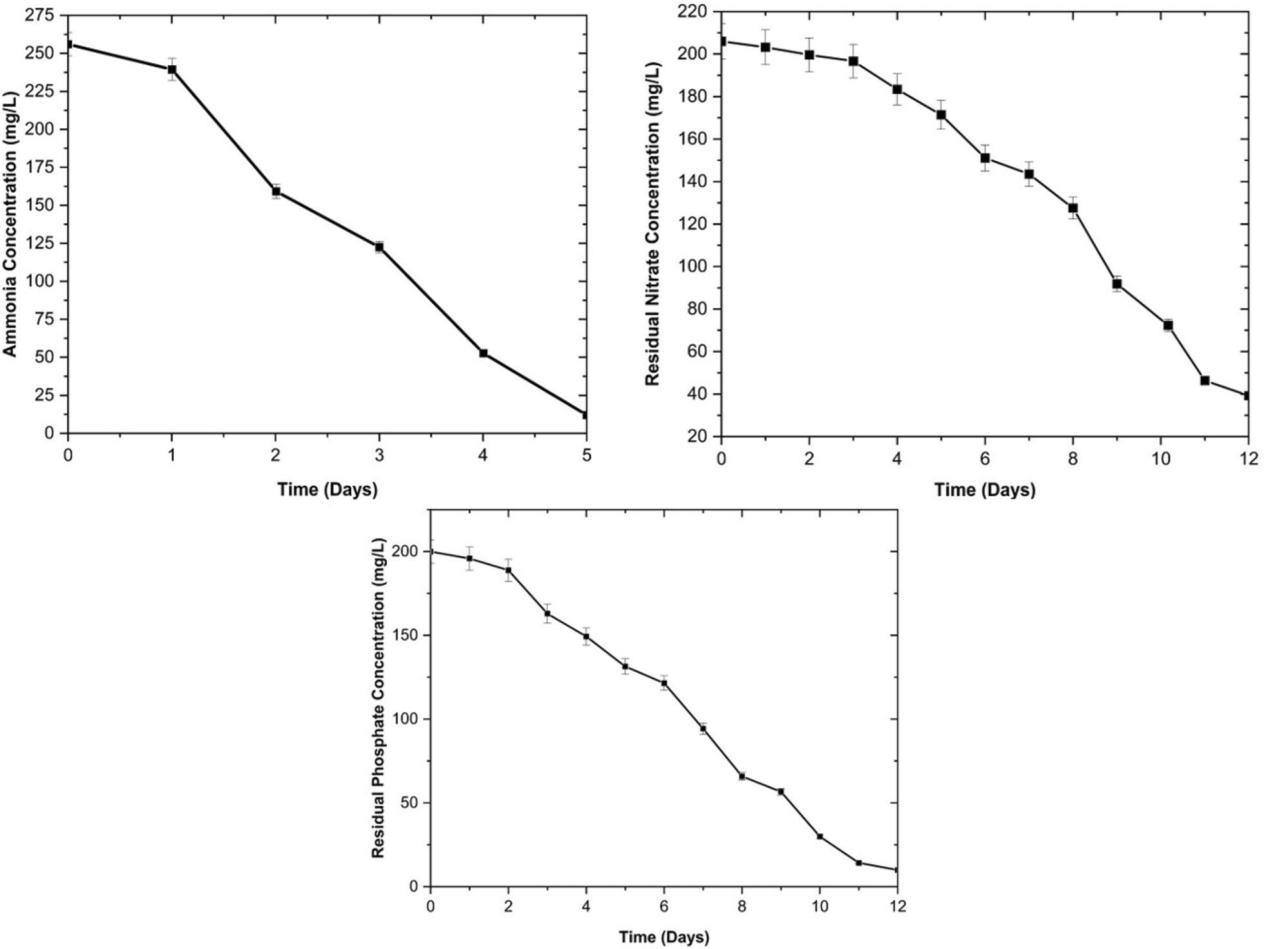
Nutrient Uptake profile of *Chlorella thermophila* in SDWW

Dairy wastewater is rich in nutrients, particularly nitrogen and phosphorus in the form of nitrate, ammonia, and phosphate [25]. In the present study, microalgal strain *Chlorella thermophila* showed a high removal efficiency of 90–95% for each of the individual nutrient components, i.e., ammonia, nitrate, and phosphate as depicted in Fig. 4. The results are consistent with previous studies where other microalgal strains, such as *Chlorella sorokiniana*, have been grown in dairy wastewater and utilized nitrate and phosphate with a removal efficiency of 67% and 73%, respectively [31]. Further investigation revealed that the microalgal strain exhibited a preference for ammonia utilization initially, while the consumption rate of nitrate was relatively slow during the initial days of the experiment. This observation aligns with previous studies that have reported microalgae's preference for assimilating ammonia over nitrate. As the ammonia in the medium is depleted, the uptake of nitrate intensifies as the microalgae adapt to the nutrient availability [22, 38].

Nitrogen and phosphorus play crucial roles in the biochemical composition and metabolic activity of microbial cells, including microalgae. Nitrogen plays a vital role as a constituent of amino acids, which serve as fundamental building blocks for proteins. Consequently, nitrate and other forms of inorganic nitrogen undergo assimilation into amino acids through diverse pathways. Microalgae typically take up inorganic nitrogen compounds like nitrate, nitrite, and ammonia that are present within the growth environment [39]. The assimilation of inorganic nitrogen can occur in any of its three prevalent forms—nitrate, nitrite, and ammonium—by traversing the plasma membrane of the cells and subsequently undergoing reduction of the oxidized nitrogen. Similarly, phosphorus holds a crucial position within algal cells due to its involvement in the synthesis of adenosine triphosphate (ATP) and the reduction of nicotinamide adenine dinucleotide phosphate (NADP +), which serves as a pivotal driver for energy-related cellular processes in living organisms [40]s. Consequently, the requirement for phosphorus by microalgae is satisfied through the utilization of phosphate present in the growth medium. The elemental analysis of the dried biomass of the microalgal strain was conducted using a CHNS elemental analyzer, and the results are presented in Table 1. The study revealed that the biomass cultivated in dairy wastewater exhibited higher contents of carbohydrates, proteins, and lipids compared to the biomass cultivated in the inorganic medium. This finding suggests that dairy wastewater provides an ideal medium for the mass cultivation of microalgal biomass, as shown in Table 2. The results show that the evaluated microalgae strains can effectively remove nitrate and can be potentially employed in a treatment process. It offers a holistic approach to wastewater treatment and resource recovery by converting waste nutrients into valuable protein, lipid, and carbohydrate-rich biomass, this process contributes to the circular economy and reduces the environmental impact of dairy wastewater discharge.

**Table 1:** Elemental analysis of *Chlorella thermophila* cultivated in different medium

Element	SDWW	BG-11
C	47.51	41.72
N	9.21	6.04
H	7.04	6.91
S	1.96	1.21

**Table 2:** Biochemical Characteristics of *Chlorella thermophila* cultivated in different medium

	BG-11	SDWW
Carbohydrate (%)	33.2 ± 1.3	32.65 ± 1.9
Protein (%)	36.13 ± 2.1	37.29 ± 1.6
Lipid (%)	21.84 ± 0.5	27.5 ± 1.2
Bioenergy(kJ)	1945.8 ± 1.6	2169.34 ± 2.7

### Analysis of antagonistic assay of the microalgal biomass extracts against plant pathogenic bacteria

**Fig. 5:**
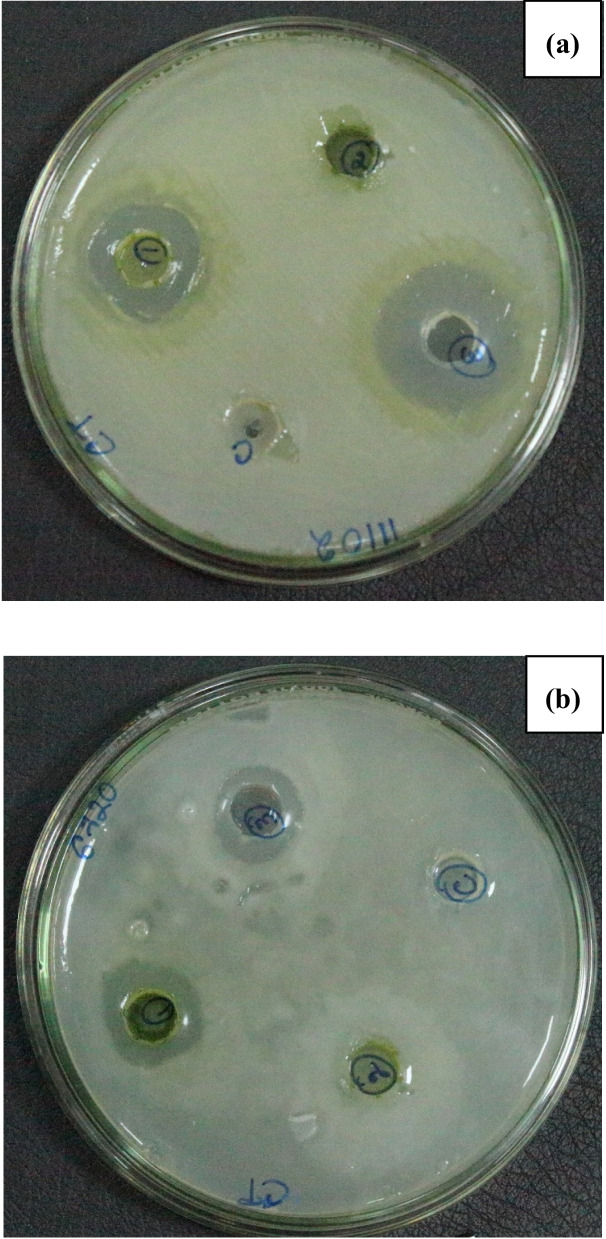
Well Diffusion Assay of *Chlorella thermophila* extract against the plant pathogen (**a**) *Xanthomonas oryzae sps oryzae* (MTCC 11102) and (**b**) *Pantoea agglomerans* (MTCC 6720)

The antagonistic effect of the microalgal biomass extract of the strain *Chlorella thermophila* was investigated against the phytopathogens *Pantoea agglomerans* (MTCC 6720) and *Xanthomonas oryzae pv. oryzae* (MTCC 11102). The bacterial strains were reported to be the causal agents for bacterial leaf blight disease in rice plants. The microalgae extract demonstrated significant inhibition towards these bacterial strains with an inhibitory index of 14% and 18% for *Pantoea agglomerans*, and *Xanthomonas oryzae pv. oryzae* respectively, as observed in the well-diffusion assay (Fig. 5). Choice of organic solvent plays a vital role in extraction of the bioactive compound from the mixture. In the present study, several organic solvents were been used for the extraction of antimicrobial compounds from the microalgae biomass extract. However, successful antimicrobial activity has been observed in case of extraction using methanol.

The GC–MS profiling of the microalgal biomass summarize around 10 chemical compounds out of which a few compounds were previously been reported for their significant bioactivity. Some of the identified compounds with significant bioactivity included N-hexadecanoic acid, 6,9-octadecadienoic acid, methyl ester, Neophytadiene, Undecanoic acid, 10-methyl-, methyl ester; 9,12-octadecadienoic acid (z,z)- Linoleic acid, Z,z-6,28-heptatriactontadien-2-one, 5,8,11-heptadecatrien-1-ol (Table 3).

**Table 3:** GC–MS profiling of *Chlorella thermophila* biomass extract

Name of the compound	Retention time	Molecular weight
N-hexadecanoic acid	29.798	256
6,9-octadecadienoic acid, methyl ester	31.519	294
Neophytadiene	31.709	278
Undecanoic acid, 10-methyl-, Methyl Ester	29.043	214
Linoleic acid	31.439	280
Z,Z-6,28-heptatriactontadien-2-one	27.622	530
5,8,11-heptadecatrien-1-ol	28.428	250

Neophytadiene is a naturally occurring hydrocarbon, specifically a diene, with the chemical formula C_19_H_32_. It is a colourless liquid that belongs to the family of acyclic sesquiterpenes, which are compounds composed of three isoprene units. It is an unsaturated fatty acid that is commonly found in a variety of natural sources, including certain plants, algae, and bacteria. It can be synthesized from phytol, which is a diterpene alcohol that is a component of chlorophyll. In addition to its industrial applications, neophytadiene has also been studied for its potential therapeutic properties. It has been found to have anti-inflammatory, anti-tumor, and anti-microbial effects in vitro, although further research is needed to determine its efficacy and safety in humans [41]. Studies have shown that neophytadiene exhibits antimicrobial activity against a range of bacterial strains, including gram-positive bacteria like *Staphylococcus aureus* and *Bacillus subtilis*, as well as gram-negative bacteria like *Escherichia coli* and *Pseudomonas aeruginosa* [42]. Neophytadiene has also been found to have antifungal activity against various fungal strains, including *Candida albicans, Aspergillus niger, and Trichophyton mentagrophytes* [43]. The exact mechanism of action of neophytadiene's antimicrobial activity is not yet fully understood, but it is believed to be due to its ability to disrupt the cell membrane of microorganisms or inhibit certain enzymes or metabolic pathways [42]. Further research is needed to explore the potential of neophytadiene as a natural antimicrobial agent and its possible use in biopesticide applications.

Another prominently found compound is linoleic acid, a polyunsaturated omega-6 fatty acid with the chemical formula C_18_H_32_O_2_. It is an essential fatty acid that plays an important role in human health, and it has potential nutraceutical applications. Linoleic acid plays a crucial role in the structure and function of cell membranes, and it is involved in various physiological processes such as inflammation, blood clotting, and immune function. One of the main nutraceutical applications of linoleic acid is its ability to reduce the risk of cardiovascular disease [44]. Linoleic acid is known to lower LDL cholesterol levels, which is the "bad" cholesterol that can build up in arteries and lead to heart disease. It has also been shown to have a positive effect on blood pressure and inflammation, both of which are risk factors for cardiovascular disease [45]. Another potential nutraceutical application of linoleic acid is its role in skin health. Linoleic acid is an important component of the skin barrier, and a deficiency in linoleic acid has been linked to skin disorders such as atopic dermatitis and acne. Supplementation with linoleic acid has been shown to improve skin hydration, elasticity, and barrier function. Other potential nutraceutical applications of linoleic acid include its role in improving immune function, reducing inflammation, and supporting brain health.

Another value-added product that has been detected in abundance in the biomass extract is Cis-9-eicosen-1-ol. Also known as nervonic acid or 24:1 omega-9, is a monounsaturated fatty alcohol with the chemical formula C_20_H_40_O [46]. Nervonic acid has been studied for its potential health benefits, particularly in relation to neurological and cognitive health. It is a key component of myelin, the fatty substance that surrounds and insulates nerve fibers in the brain and spinal cord [47]. Nervonic acid has been found to play a role in maintaining the structure and function of myelin, and a deficiency in nervonic acid has been linked to certain neurological disorders, such as multiple sclerosis and Alzheimer's disease [48]. In addition to its role in neurological health, nervonic acid has been found to have potential benefits for cardiovascular health. One study found that supplementation with nervonic acid improved lipid profiles and reduced inflammation in individuals with high cholesterol levels [46].

The simulated wastewater seems to contain an ample amount of micro- and macronutrients. The analysis of fatty acid methyl ester (FAME) spectra revealed the presence of both saturated and unsaturated fatty acids (USFA) in varying amounts in the microalgal biomass. The major fatty acids detected included C-16:0, C-16:1, C-18:0, C-18:1, C-18:2, and C-18:3. The microalgal biomass exhibited a high proportion of long-chain fatty acids (LCFA), which are desirable for biodiesel production. Prominent LCFA identified were myristic acid, palmitic acid, palmitoleic acid, stearic acid, oleic acid, and linoleic acid. Among the saturated fatty acids, palmitic acid was found in relatively higher quantities compared to stearic acid and myristic acid. These findings suggest that the microalgal biomass holds promise as a potential feedstock for biodiesel production due to its favorable fatty acid composition [28].

### Hydrothermal liquefaction for biofuel production from residual biomass after extraction

In this study, the biomass extracted for biopesticide production was utilized for the production of biocrude. The biocrude yield obtained was 28%, which is comparable to previous studies using various feedstocks under similar experimental conditions, including temperature and residence time [49, 50]. The aqueous phase accounted for 50% (w/w) of the total content, while the solid residue constituted 17% (w/w). The conversion rate was determined to be 86.4% (w/w). Previous research on hydrothermal liquefaction using microalgal biomass as feedstock has reported biocrude yields ranging between 18 and 31% (w/w) at a reaction temperature of 350 °C and a residence time of 15 min [29, 51]. The obtained biocrude was further characterized to evaluate its potential as a biofuel. Elemental composition analysis revealed that the biocrude primarily consisted of carbon (74.4% w/w), hydrogen (10.64% w/w), and oxygen (11.15% w/w). Nitrogen (3.56% w/w) and sulfur (0.25% w/w) were present in significantly lower proportions. Additionally, the biocrude exhibited an increased higher heating value (HHV) of 38.19 MJ/kg, which was 91.12% higher than that of the feedstock (19.98 MJ/kg). Compared to previous reports, the elemental composition of the obtained biocrude was found to be superior, highlighting its potential as a biofuel feedstock even after biopesticide extraction. This suggests that the biocrude retains favorable levels of key elements such as carbon, hydrogen, oxygen, nitrogen, and sulfur, which are essential for the efficient conversion of biomass into biofuels. The presence of these elements at desirable levels suggests that the biocrude has a high energy content and can serve as a promising feedstock for biofuel production. The superior elemental composition of the biocrude is a positive indication for its suitability as a biofuel feedstock, as it means that it contains the necessary components for effective and efficient conversion processes. Therefore, the findings of this study confirm that *Chlorella thermophila* is a promising candidate for biocrude production, even after the extraction of biopesticide from the biomass. This finding is significant because the extraction of biopesticides might alter the chemical composition of the biomass, potentially affecting its prospect as a biofuel source. However, the analysis shows that the biocrude maintains its advantageous elemental composition even after biopesticide extraction, reaffirming its potential as a viable and sustainable source of biofuels.

### Proposed biorefinery model

**Fig. 6:**
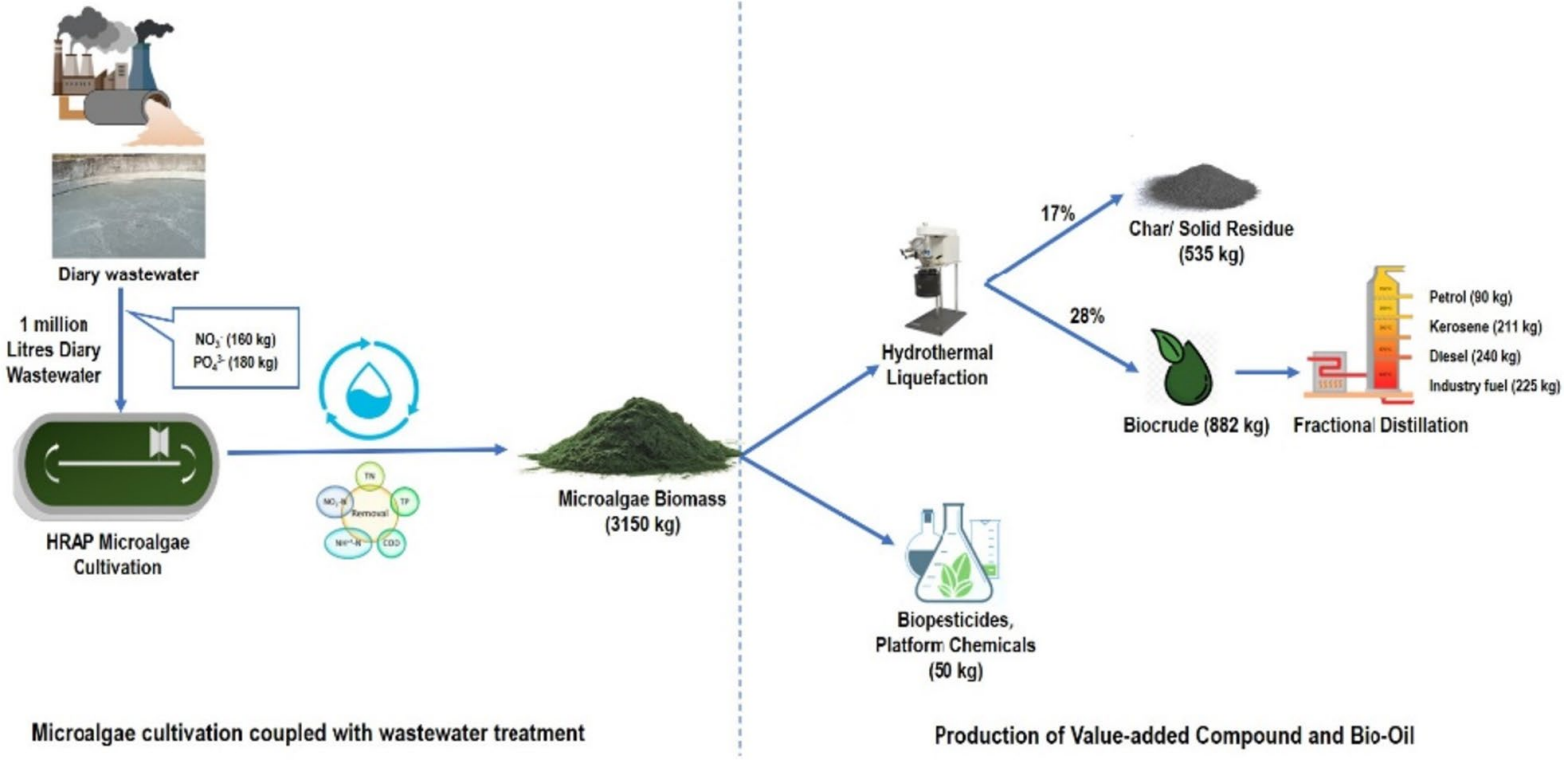
Proposed Biorefinery model

A microalgal biorefinery process represents an integrated approach that encompasses both upstream and downstream operations, aiming for economically efficient production and the effective conversion of microalgal biomass into biofuel. This innovative concept combines biorefinery principles with wastewater treatment, thus maximizing the utilization of algal biomass and minimizing overall waste generated throughout the process [52]. The primary objective of this research is to extract a variety of valuable co-products from harvested microalgal biomass and formulate a biorefinery model that can be readily applied on a commercial scale. The development of this biorefinery model (depicted in Fig. 6) takes into account experimental findings, specifically the optimized levels of nitrogen (N) and phosphorus (P) determined in the earlier study. In this modelling process, a baseline of 1 million litres of dairy waste water was used for microalgae cultivation, with corresponding nutrient inputs of 160 kg of nitrate and 180 kg phosphate respectively. Cultivating it in HRAP raceways, it will generate 3150 kg of microalgae biomass of *Chlorella thermophila* which will generate around 50 kg of the biopesticide and other platform chemicals. The extracted biomass can further be subjected to hydrothermal liquefaction which will yield around 882 kg of biocrude and 535 kg of biochar as the solid residue. The biocrude on further processing by fractional distillation could generate around 90 kg of petrol, 211 kg of kerosene, 240 kg of diesel and 225 kg of industrial fuel. The biochar produced could further be used for the remediation of CECs from the water bodies or industrial effluents.

## Conclusion

The utilization of dairy wastewater as a growth medium for microalgae cultivation shows great promise in reducing freshwater dependency. Among the microalgal strains, *Chlorella thermophila* (CT) exhibits the ability to recover nutrients from the wastewater, thus promoting a sustainable approach in the field of bioremediation. This study sheds light on the strain's potential not only in remediating dairy wastewater by utilizing its nutrients but also in offering plant protection against harmful phytopathogens. Assessing the biopesticide potential of CT reveals interesting results, as it demonstrates the ability to inhibit the growth of both *Xanthomonas oryzae sp oryzae* and *Pantoea agglomerans*, two phytopathogens. The obtained phytochemicals hold high value and can be applied in various practical applications. Implementing microalgae as biopesticides presents a promising alternative to replace harmful chemicals without compromising crop yield. Furthermore, this study confirms that *Chlorella thermophila* remains a promising candidate for bioenergy production, even after extracting biopesticides from the biomass. Thus, the potential of both *Chlorella thermophila* biomass and biopesticide-extracted biomass as high-value bioenergy feedstocks is confirmed, enabling the production of value-added co-products. These findings contribute to the realization of a self-sustainable, energy-efficient, and cost-effective microalgae biorefinery system. Further optimization and scale-up studies are required to assess the techno-economic feasibility of a zero-waste biorefinery system.

### Supplementary Information

**Additional file 1.** Supplementary Information.

## Data Availability

All data generated or analysed during this study are included in this published article and in the Supplementary files.
