# Supplementary material for: Valorization of Chlorella thermophila biomass cultivated in dairy wastewater for biopesticide production against bacterial rice blight: a circular biorefinery approach
Source: BMC Plant Biol. 2023 Dec 15;23:644. doi: 10.1186/s12870-023-04579-z (PMC10722807; doi:10.1186/s12870-023-04579-z)
Supplement: Supplementary file 1 — Additional file 1. Supplementary Information. [file 12870_2023_4579_MOESM1_ESM.docx]

**Supplementary Information:**

Valorization of *Chlorella thermophila* biomass cultivated in dairy wastewater for biopesticide production against bacterial rice blight: A circular biorefinery approach

Satya Sundar Mohanty ^a, c^, Kaustubha Mohanty ^a, b*^

*^a^ School of Energy Sciences and Engineering, Indian Institute of Technology Guwahati, Assam, India*

*^b^ Department of Chemical Engineering, Indian Institute of Technology Guwahati, Assam, India*

*^c^ Department of Biotechnology, Karunya Institute of Technology and Sciences, Coimbatore, Tamil Nadu, India*

* Corresponding author: E-mail: kmohanty@iitg.ac.in (K. Mohanty)

**Table S1:** BG 11 medium composition for microalgae [1].

| Nutrient | Concentration (mg L^-1^) |
| --- | --- |
| NaNO_3_ | 1500.00 |
| K_2_HPO_4_ | 40.00 |
| MgSO_4_.7H_2_O | 75.00 |
| CaCl_2_.7H_2_O | 36.00 |
| Citric acid | 6.00 |
| Na_2_CO_3_ | 20.00 |
| Na_2_EDTA | 1.00 |
| C_6_H_8_FeNO_7_ | 6.00 |
| H_3_BO_3_ | 2.860 |
| MnCl_2_.4H_2_0 | 1.810 |
| ZnSO_4_.7H_2_O | 0.222 |
| Na_2_MoO_4_.2H_2_O | 0.390 |
| CuSO_4_.5H_2_O | 0.079 |
| Co(NO_3_)_2_.6H_2_O | 0.0494 |

**Table S2**: Statistical analysis on cells growth data

| **Logistic growth** | **BG 11 Medium** | **SDWW** |
| --- | --- | --- |
| Best-fit values |  |  |
| YM | 2.396 | 2.978 |
| Y0 | 0.2169 | 0.2389 |
| k | 0.2628 | 0.2689 |
| X_int_ | 3.805 | 3.719 |
| Goodness of Fit |  |  |
| Degrees of Freedom | 10 | 10 |
| R squared | 0.9991 | 0.9957 |
| Adjusted R squared | 0.9989 | 0.9948 |
| Sum of Squares | 0.002625 | 0.01973 |
| Sy.x | 0.01620 | 0.04442 |
| RMSE | 0.01479 | 0.04055 |
| AICc | -97.60 | -71.37 |
| Normality of Residuals |  |  |
| Anderson-Darling (A2*) | 0.4264 | 0.2662 |
| P value | 0.2661 | 0.6278 |

[1] R.Y. Stanier, R. Kunisawa, M. Mandel, G. Cohen-Bazire, Purification and properties of unicellular blue-green algae (order Chroococcales)., Bacteriol. Rev. 35 (1971) 171–205.
